# Biological characterization of expression quantitative trait loci (eQTLs) showing tissue-specific opposite directional effects

**DOI:** 10.1038/s41431-019-0468-4

**Published:** 2019-07-11

**Authors:** Akira Mizuno, Yukinori Okada

**Affiliations:** 1 Faculty of Medicine, Osaka University, Suita, 565-0871 Japan; 2 Department of Statistical Genetics, Graduate School of Medicine, Osaka University, Suita, 565-0871 Japan; 3 Laboratory of Statistical Immunology, Immunology Frontier Research Center (WPI-IFReC), Osaka University, Suita, 565-0871 Japan

**Keywords:** Gene expression, Gene regulatory networks

## Abstract

Interpreting the susceptible loci documented by genome-wide association studies (GWASs) is of utmost importance in the post-GWAS era. Since most complex traits are contributed by multiple tissues, analyzing tissue-specific effects of expression quantitative trait loci (eQTLs) is a promising approach. Here we describe “opposite eQTL effects”, i.e., gene expression effects of eQTLs that are in the opposite direction between different tissues, as the biologically meaningful annotations of genes and genetic variants for understanding the GWAS loci. The genes and single-nucleotide polymorphisms (SNPs) associated with the opposite eQTL effects (*opp-multi-eQTL-Genes* and *opp-multi-eQTL-SNPs*) were extracted from the largest eQTL database provided by the Genotype-Tissue Expression (GTEx) project (release version 7). The opposite eQTL effects were detected even between closely related tissues such as cerebellum and brain cortex, and a significant proportion of the genes having eQTLs were annotated as the *opp-multi-eQTL-Genes* (2,323 out of 31,212; 7.4%). The *opp-multi-eQTL-SNPs* showed locational enrichment at the transcription start site and also possible involvement of epigenetic regulation. The biological importance of the opposite eQTL effects was also assessed using the SNPs reported in GWASs (*GWAS-SNPs*), which demonstrated that a high proportion of the *opp-multi-eQTL-SNPs* are in linkage disequilibrium with the *GWAS-SNPs* (2,498 out of 9,290; 26.9%). Based on the results, the opposite eQTL effects can be a common phenomenon in the tissue-specific gene regulation with a possible contribution to the development of complex traits.

## Introduction

Genome-wide association studies (GWASs) have documented thousands of susceptible loci, mostly single-nucleotide polymorphisms (SNPs), that may have causal effects on the complex traits such as height, body mass index, and disease prevalence [1]. However, most of the susceptible loci were found in non-coding regions of the genome, and the biological mechanisms underlying the associations are poorly understood [2]. Therefore, in this so-called post-GWAS era, collecting biologically meaningful annotations of genes and genetic variants is essential for interpreting the results of GWASs [3].

Building functional annotation database started as the large-scale projects in 2000s, represented by FANTOM [4], ENCODE [5], and Roadmap Epigenomics [6] projects. Collecting higher-order functional information, e.g., 4D-Nucleome project [7], is also undergoing. The Genotype-Tissue Expression (GTEx) project was launched in 2010 as one of those large-scale functional annotation projects, and it is focused on mapping expression quantitative trait loci (eQTLs) for various tissues to elucidate the genetic variants underlying altered gene expression [8, 9]. The change of gene expression levels is such a fundamental trait influencing the functions of cells and tissues that the large eQTL catalog obtained by the GTEx project would be a strong basis for interpreting the GWAS loci [10]. Most importantly, revealing the tissue-specific pattern of gene regulation would give a clue to understand the biological causes of complex traits involved by multiple tissues [11]. Since the GTEx project produced the largest eQTL database by analyzing 48 tissues from 620 donors (release version 7), it gives us a valuable opportunity to explore tissue-specific effects of eQTLs as the biologically meaningful annotations of genes and genetic variants.

In the context of the tissue-specific eQTL effects, it is interesting to focus on the phenomenon that the direction of eQTL effects on a given gene is discordant depending on the tissue type, because those eQTL effects could be the factors distinguishing characteristics of the tissues [12]. The characteristic difference would be larger and hence be relevant to the development of biological traits, when the discordance is detected as “opposite eQTL effects”, i.e., the gene expression effects of eQTLs are in the opposite direction between different tissues. Discovery of SNPs with such opposite eQTL effects was previously reported in GWASs in conjunction with eQTL studies [13, 14], indicating their possible contribution to the complex traits including disease prevalence.

However, due to the significant limitation of the number of tissue types and sample size available for the eQTL analysis, reports on the opposite eQTL effects are still rare compared to the enormous number of eQTL studies, and their statistical aspects are poorly documented despite their possible importance in understanding the results of GWASs. Moreover, analyzing the opposite eQTL effects based on the primary, or most significant, eQTL signals with the linkage disequilibrium (LD) relationship taken into account has not been performed. The most significant eQTL variants are enriched at the transcription start site (TSS) to be the most likely functional variants [15]. Therefore, for the accurate assessment of the discordance of the eQTL effects on a given gene between different tissues, it is necessary to compare the direction of the primary eQTL signals of SNPs in LD.

We here report the statistics of the genes and SNPs associated with the opposite eQTL effects in the latest version of the GTEx database via full evaluation of the LD relationship between SNPs with the most significant eQTL signal in each tissue. Biological properties of the opposite eQTL effects were assessed by enrichment analysis on the distance from TSS and epigenetic annotations. Their biological importance was also investigated by LD analysis against SNPs reported in the GWAS catalog [16].

## Materials and methods

### Extraction of gene/SNP pairs with the most significant eQTL signal in each tissue from the GTEx database

The significant variant-gene association results of the single-tissue *cis*-eQTL analysis for 48 tissues were downloaded from the GTEx portal website (release version 7, file name: GTEx_Analysis_v7_eQTL.tar.gz;https://www.gtexportal.org/). The significance level threshold for eQTL effects was reported in the original paper of the GTEx project [9]. dbSNP-based rsID was used for description of SNPs. For the rsIDs that appeared in this article, their corresponding description based on the human genome reference sequence (GRCh37) is summarized in Supplementary Table S1. From the downloaded GTEx data, indel variants, multi-allelic SNPs, and SNPs not included in the 1000 genomes project results (phase 3, version 5; http://www.internationalgenome.org/) [17] were removed. Genes that have at least one SNP with an eQTL signal in one or more tissues, denoted by *eQTL-Genes*, were extracted as *eQTL-Gene* and SNP pairs, resulting in 6,895,474 pairs (31,212 genes, 2,842,590 SNPs). From the *eQTL-Gene* and SNP pairs, SNPs that showed the smallest *p*-value in each tissue, denoted by *top-eQTL-SNPs*, were then extracted as *eQTL-Gene* and *top-eQTL-SNP* pairs, resulting in 232,457 pairs (31,212 genes, 210,878 SNPs). In the case that there were more than one *top-eQTL-SNPs* in a single tissue, i.e., multiple SNPs showed the identical smallest *p*-value due to the strong LD relationship, the lexicographically first rsID was designated as the *top-eQTL-SNP* for the tissue.

### Analysis of directional difference of eQTL effects between different two tissues

Between different two tissues, the directional difference of primary eQTL effects was assessed based on a 2 × 2 analysis table, as summarized in Supplementary Fig. S1. The analysis table was made for each *eQTL-Gene* between each tissue pair (35,207,136 analysis tables in total for 31,212 *eQTL-Genes* and 1128 tissue pairs from 48 tissues). Let SNP-*x* be the *top-eQTL-SNP* of a given *eQTL-Gene* in tissue-*x*, and *β*_*xy*_ be the effect size of SNP-*x* in tissue-*y* (if SNP-*x* is not significantly detected in tissue-*y*, *β*_*xy*_ was set as 0). Only the cases that the given *eQTL-Gene* has SNP(s) with a significant eQTL signal in both tissues in the pair (i.e., *β*_*xx*_  ≠ 0 ∩ *β*_*yy*_ ≠ 0) were moved into the next analysis (3,540,453 analysis tables). When at least one of SNP-*x* and SNP-*y* is significant in both tissues (i.e., *β*_*xy*_ ≠ 0 ∪ *β*_*yx*_ ≠ 0), the directional difference of the eQTL effects between the tissue pair can be discussed. Therefore, the cases of (i) SNP-*x* and SNP-*y* are identical (i.e., *β*_*xx*_ = *β*_*yx*_ and *β*_*xy*_ = *β*_*yy*_) and (ii) SNP-*x* and SNP-*y* are not identical but at least one of them is significant in both tissues were selected (2,488,564 analysis tables). The directional difference of the eQTL effects between the tissue pair was first determined by the sign of *β*_*xx*_ × *β*_*xy*_ and *β*_*yx*_ × *β*_*yy*_, and the cases of *β*_*xx*_ × *β*_*xy*_ ≤ 0 ∩ *β*_*yx*_ × *β*_*yy*_ ≤ 0 were considered as opposite direction. Next, the *r*^*2*^ coefficient of the LD between SNP-*x* and SNP-*y* were calculated by PLINK software (version 1.9; https://www.cog-genomics.org/plink2) [18] using the 1000 genomes project data as the reference panel (EUR population), and the analysis tables were divided into four groups (**1a**, **1b**, **2a**, and **2b** shown in Fig. S1) based on *r*^*2*^ threshold of 0.8 (if SNP-*x* and SNP-*y* are identical, *r*^*2*^ was set as 1). The *eQTL-Genes* and *top-eQTL-SNPs* in group **1a** or **2a** (any direction, *r*^*2*^ > 0.8) were designated as *multi-eQTL-Genes* and *multi-eQTL-SNPs* (113,274 pairs consisting of 17,192 genes and 101,621 SNPs), and those in group **1a** (opposite direction, *r*^*2*^ > 0.8) were designated as *opp-multi-eQTL-Genes* and *opp-multi-eQTL-SNPs* (9541 pairs consisting of 2323 genes and 9290 SNPs).

### Clustering analysis of tissue types based on opposite eQTL effect fractions

In each tissue pair from 48 tissues (1128 pairs in total), *β*-values of all SNPs included in the significant eQTL analysis result dataset were compared between the tissues, and the proportion of the number of SNPs showing opposite directional *β*-values was calculated. (Note: This analysis was focused not only on *top-eQTL-SNPs* but on all SNPs showing significant eQTL signals. SNPs that are not significant in both tissues of the pair were omitted from the analysis.) Clustering the tissue types based on the opposite eQTL effect fractions was conducted by the R software (version 3.3.0).

### Distribution analysis of the distance from TSS

Distance from TSS was retrieved from the GTEx database for all SNPs in each type of gene and SNP pairs (*eQTL-Gene* and SNP with any eQTL signal, *eQTL-Gene* and *top-eQTL-SNP*, *multi-eQTL-Gene* and *multi-eQTL-SNP*, *opp-multi-eQTL-Gene* and *opp-multi-eQTL-SNP*). A subset of the *multi-eQTL-Gene* and *multi-eQTL-SNP* pairs was randomly sampled so that the null distribution of the number of tissues in which a significant eQTL signal of the same gene and SNP pair was detected (i.e., the number of sharing tissues) was adjusted between the *multi-eQTL-SNPs* and the *opp-multi-eQTL-SNPs*. The density distribution of the distance from TSS for each type of SNPs was analyzed by the R software (version 3.3.0). The significance level of the TSS enrichment in the *opp-multi-eQTL-SNPs* compared to the adjusted *multi-eQTL-SNPs* was evaluated based on kurtosis of the density distribution by 10,000-time random sampling.

### Epigenetic annotation analysis on histone modification and DNase sensitivity

To adjust the distance from TSS, a subset of the *multi-eQTL-SNPs* (TSS-distance-adjusted *multi-eQTL-SNPs*) was generated by random sampling so that the null distribution of the distance form TSS was matched with that of the *opp-multi-eQTL-SNPs*. Specifically, the *opp-multi-eQTL-Gene* and *opp-multi-eQTL-SNP* pairs in each 1-kbp window from −1000 to +1000 kbp TSS distance were counted, and the same number of pairs were randomly sampled from the *multi-eQTL-Gene* and *multi-eQTL-SNP* pairs for each window to generate the adjusted dataset of *multi-eQTL-SNPs*. The SNP annotation dataset was downloaded from the HaploReg v4.1 website (accessed 2019/02/01; file name: haploreg_v4.0_20151021.vcf.gz; https://pubs.broadinstitute.org/mammals/haploreg/data/) [19], which includes epigenetic annotations from the Roadmap Epigenomics project on up to 127 cell lines [6]. The annotations were histone modification states (H3K4me1, H3K4me3, H3K9ac, and H3K27ac) and sensitivity to cleavage by DNase. For enrichment analysis of these epigenetic annotations, the fraction of SNPs with each epigenetic annotation in each type of SNPs (*multi-eQTL-SNPs*, TSS-distance-adjusted *multi-eQTL-SNPs*, or *opp-multi-eQTL-SNPs*) was calculated for each of the cell lines in which the epigenetic annotation data were available. The mean fraction across the available cell lines was used as a surrogate to investigate the enrichment. The random generation of the TSS-distance-adjusted *multi-eQTL-SNPs* was repeated by 10,000 times to provide 95% confidence interval.

### Assessment of LD relationship with the SNPs reported in the GWAS catalog

The summary information about SNPs reported in GWASs (*GWAS-SNPs*) was downloaded from the GWAS catalog website (accessed 2019/03/06; file name: gwas_catalog_v1.0.2-associations_e93_r2019-01-31.tsv; https://www.ebi.ac.uk/gwas/) [16], and SNPs whose reported *p*-value is less than 5 × 10^−8^ were extracted. For each type of SNPs in the GTEx database (SNPs with any eQTL signals, *top-eQTL-SNPs*, *multi-eQTL-SNPs*, TSS-distance-adjusted *multi-eQTL-SNPs*, and *opp-multi-eQTL-SNPs*), the *r*^*2*^ coefficient of the LD with the *GWAS-SNPs* was calculated by PLINK 1.9 using 1000 genomes project phase 3 data version 5 (EUR population) as the reference. The number of SNPs whose *r*^*2*^ coefficient was more than 0.8 with at least one *GWAS-SNP* was counted. The significance level of the percentage of SNPs in LD with the *GWAS-SNPs* in the *opp-multi-eQTL-SNPs* was investigated by Fisher’s exact test (compared to the *multi-eQTL-SNPs*) and 10,000-time random sampling (compared to the TSS-distance-adjusted *multi-eQTL-SNPs*). Some examples of the *opp-multi-eQTL-SNPs* that showed the LD with the *GWAS-SNP*(*s*) were chosen, and the distribution of the *β*-value and negative logarithm of *p*-value of all tested SNPs within 1 Mbp distance from TSS with respect to the position on chromosome were plotted by the R software (version 3.3.0). The *β*-value and *p*-value of all tested SNPs including SNPs that showed non-significant eQTL signals were downloaded from the GTEx portal site (release version 7, file name: GTEx_Analysis_v7_eQTL_all_associations.tar.gz; https://www.gtexportal.org/). For clarification of the directional difference of the eQTL effects between tissues, reference and alternative alleles were reassigned so that the signs of *β*-values of all tested SNPs are all positive in one of the tissues of the pair. In the other tissue, the *β*-values were plotted according to the reassigned reference and alternative alleles.

## Results

### Grouping genes and SNPs for analysis of the opposite eQTL effects

**Fig. 1:**
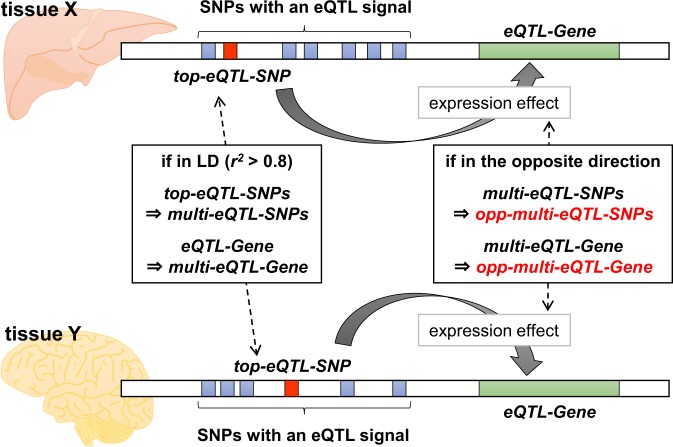
Graphical scheme for extraction of the genes and SNPs associated with opposite eQTL effects between different tissues. In each tissue, genes that have at least one SNP with a significant eQTL signal are denoted as *eQTL-Genes*, and for each *eQTL-Gene*, the SNP with the most significant eQTL signal is designated as *top-eQTL-SNP*. In a tissue pair (i.e., tissue X and tissue Y in the figure), if the linkage disequilibrium (LD) coefficient (*r*^2^) between the *top-eQTL-SNPs* is more than 0.8, the *top-eQTL-SNPs* and *eQTL-Gene* are denoted as *multi-eQTL-SNPs* and *multi-eQTL-Gene*, respectively. Subsequently, the direction of the effect sizes of the *multi-eQTL-SNPs* are compared in the tissue pair, and if the effects are in the opposite direction, the *multi-eQTL-SNPs* and *multi-eQTL-Gene* are highlighted as *opp-multi-eQTL-SNPs* and *opp-multi-eQTL-Gene*, respectively. All tissue pairs in the GTEx database release version 7 (1128 pairs from 48 tissues) are examined to extract the *opp-multi-eQTL-SNPs* and *opp-multi-eQTL-Genes*

From the GTEx database (release version 7), genes and SNPs relating to the opposite eQTL effects between different tissues were extracted stepwise, the graphical scheme of which is depicted in Fig. 1 (see Materials and methods for details). Briefly, when one or more SNPs showed an eQTL signal on the expression of a gene in either tissue, the affected gene was denoted as *eQTL-Gene*, and the SNP showing the most significant eQTL signal was denoted as *top-eQTL-SNP*. Focusing on an *eQTL-Gene* in a given tissue pair (tissue X and tissue Y in Fig. 1), if the two *top-eQTL-SNPs* are identical or in LD (*r*^*2*^ > 0.8), the *top-eQTL-SNPs* and the *eQTL-Gene* were designated as *multi-eQTL-SNPs* and *multi-eQTL-Gene*, respectively. Subsequently, if the expression effects of the *multi-eQTL-SNPs* on the *multi-eQTL-Gene* are in the opposite direction between tissue X and tissue Y, the *multi-eQTL-SNPs* and *multi-eQTL-Gene* were highlighted as *opp-multi-eQTL-SNPs* and *opp-multi-eQTL-Gene*, respectively. If the opposite direction of expression effects was detected in at least one tissue pair, the genes and SNPs were considered to be associated with the opposite eQTL effects.

Overall results of extracting each type of gene/SNP pairs from 48 tissues in the GTEx database are summarized in Table 1. The *opp-multi-eQTL-Genes* accounted for 7.4% of the *eQTL-Genes* (2,323 out of 31,212 *eQTL-Genes*), and the *opp-multi-eQTL-SNPs* accounted for 4.4% of the *top-eQTL-SNPs* (9,290 out of 210,878 *top-eQTL-SNPs*). The proportion of the *opp-multi-eQTL-Genes* was unexpectedly high when compared to those in the previous eQTL studies, in which a few proportions (approximately 1.4% of gene probes) showed opposite effect sizes between blood and four non-blood tissues [12]. This increase could be mainly due to the large number of tissue types in the GTEx database, which provided combinatorial increase of the tissue pairs to assess the discordance of eQTL effects, giving high chance to discover SNPs with the opposite eQTL effects. Based on this result, the existence of the opposite eQTL effects might have been underestimated, and the more tissue types are analyzed, the more opposite eQTL effects can be discovered.

**Table 1:** Results of extracting each type of gene/SNP pairs from the GTEx database

Type of gene and SNP pair	Number of pairs	Number of genes	Number of SNPs
*eQTL-Gene* and SNP with any eQTL signal	6,895,474	31,212	2,842,590
*eQTL-Gene* and *top-eQTL-SNP*	232,457	31,212	210,878
*multi-eQTL-Gene* and *multi-eQTL-SNP* (*r*^2^ > 0.8 in at least 1 tissue pair)	113,274	17,192	101,621
*opp-multi-eQTL-Gene* and *opp-multi-eQTL-SNP* (opposite in at least 1 tissue pair)	9,541	2,323 (7.4% of *eQTL-Genes*)	9,290 (4.4% of *top-eQTL-SNPs*)

The distribution of significance levels of the opposite eQTL effects were also investigated based on the *p*-values in each tissue pair where *multi-eQTL-SNPs* or *opp-multi-eQTL-SNPs* were detected. Since there are two *p*-values from the two tissues of a pair, *p*-value_1 was designated as the lower one, and *p*-value_2 was designated as the higher one. The distribution of negative logarithm of *p*-value_1 and 2 is shown in Supplementary Fig. S2. The difference between the 2D density maps of *multi-eQTL-SNPs* and *opp-multi-eQTL-SNPs* is also shown. The density in the *opp-multi-eQTL-SNPs* is high along the horizontal axis and low in the diagonal area compared to the density in the *multi-eQTL-SNPs*. Therefore, the significance levels of the top eQTL effects tend to deviate more largely between the two tissues of the pair that showed opposite eQTL effects. This finding could be understandable based on the possibility that the opposite eQTL effects resulted from altered gene regulation patterns depending on tissue types, the statistical detection level of which would largely vary across tissues.

### Tissue-dependent characteristics of the opposite eQTL effects

**Fig. 2:**
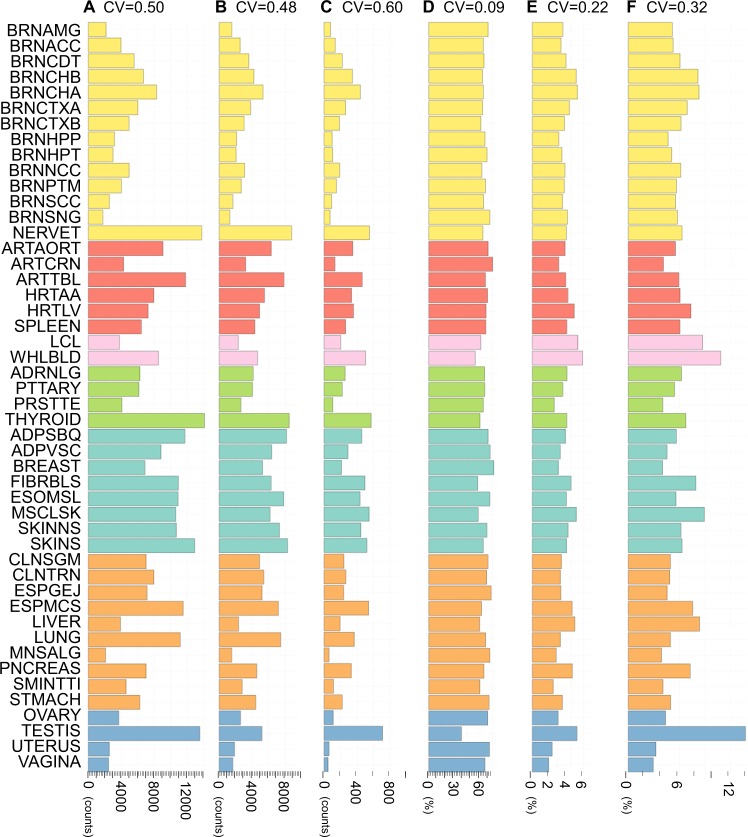
Counts and proportions of *eQTL-*, *multi-eQTL-*, and *opp-multi-eQTL-Genes* for 48 tissues in the GTEx database (release version 7). **A** Counts of *eQTL-Genes*. **B** Counts of *multi-eQTL-Genes*. **C** Counts of *opp-multi-eQTL-Genes*. **D** Proportion (%) of *multi-eQTL-Genes* in *eQTL-Genes*. **E** Proportion (%) of *opp-multi-eQTL-Genes* in *eQTL-Genes*. **F** Proportion (%) of *opp-multi-eQTL-Genes* in *multi-eQTL-Genes*. CV stands for coefficient of variation

The number of *eQTL-Genes*, *multi-eQTL-Genes*, and *opp-multi-eQTL-Genes* for each tissue are shown in Fig. 2**A**–**C**. The tissues are shown by abbreviations and categorized by the organ systems (Supplementary Table S2). The number of each type of genes varied in different tissues (coefficient of variation (CV) = 0.48–0.60). This variation was mainly caused by the difference of the sample sizes obtained for each tissue, because the number of identified *eQTL-Genes* in a tissue increases linearly with its sample size [20]. To remove the sample size bias, the proportion of the *multi-eQTL-Genes* (=*multi-eQTL-Genes*/*eQTL-Genes*) and *opp-multi-eQTL-Genes* (=*opp-multi-eQTL-Genes*/*eQTL-Genes* and *opp-multi-eQTL-Genes*/*multi-eQTL-Genes*) were calculated and shown in Fig. 2**D**–**F**. There was negative correlation between the proportion of *multi-eQTL-Genes* and that of *opp-multi-eQTL-Genes* (Pearson’s correlation coefficient *r* = −0.54 between *multi-eQTL-Genes*/*eQTL-Genes* (**D**) and *opp-multi-eQTL-Genes*/*eQTL-Genes* (**E**), and *r* = −0.81 between *multi-eQTL-Genes*/*eQTL-Genes* (**D**) and *opp-multi-eQTL-Genes*/*multi-eQTL-Genes* (**F**)).

Since the *multi-eQTL-Genes* are the genes whose most significant eQTL is shared by multiple tissues, the index of *multi-eQTL-Genes*/*eQTL-Genes* could be interpreted as the similarity of the gene regulation pattern among the tissues. Therefore, its negatively correlated index, *opp-multi-eQTL-Genes*/(*multi-*)*eQTL-Genes*, could indicate the difference of the gene regulation pattern compared to the other tissues. In addition, the *opp-multi-eQTL-Genes*/(*multi-*)*eQTL-Genes* can be used as a sensitive index to catch the gene regulation uniqueness, because the CV of *opp-multi-eQTL-Genes*/(*multi-*)*eQTL-Genes* (0.22 and 0.32) was larger than that of *multi-eQTL-Genes*/*eQTL-Genes* (0.09). Testis showed the smallest value of *multi-eQTL-Genes*/*eQTL-Genes* (38.1%) and the highest value of *opp-multi-eQTL-Genes*/*multi-eQTL-Genes* (13.7%), which would be reasonable because testis clearly showed different gene expression pattern, e.g., testis expressed the largest number of genes and many of them were uniquely expressed. Following to testis, high value of *opp-multi-eQTL-Genes*/*multi-eQTL-Genes* was detected for cerebellar tissues (BRNCHB; 8.1% and BRNCHA; 8.3%), blood cells (LCL; 8.7% and WHLBLD; 10.8%), fibroblasts (FIBRBLS; 7.9%), skeletal muscle (MSCLSK; 8.9%), and some gastrointestinal tissues (ESPMCS; 7.5%, LIVER; 8.3%, and PNCREAS; 7.2%).

**Fig. 3:**
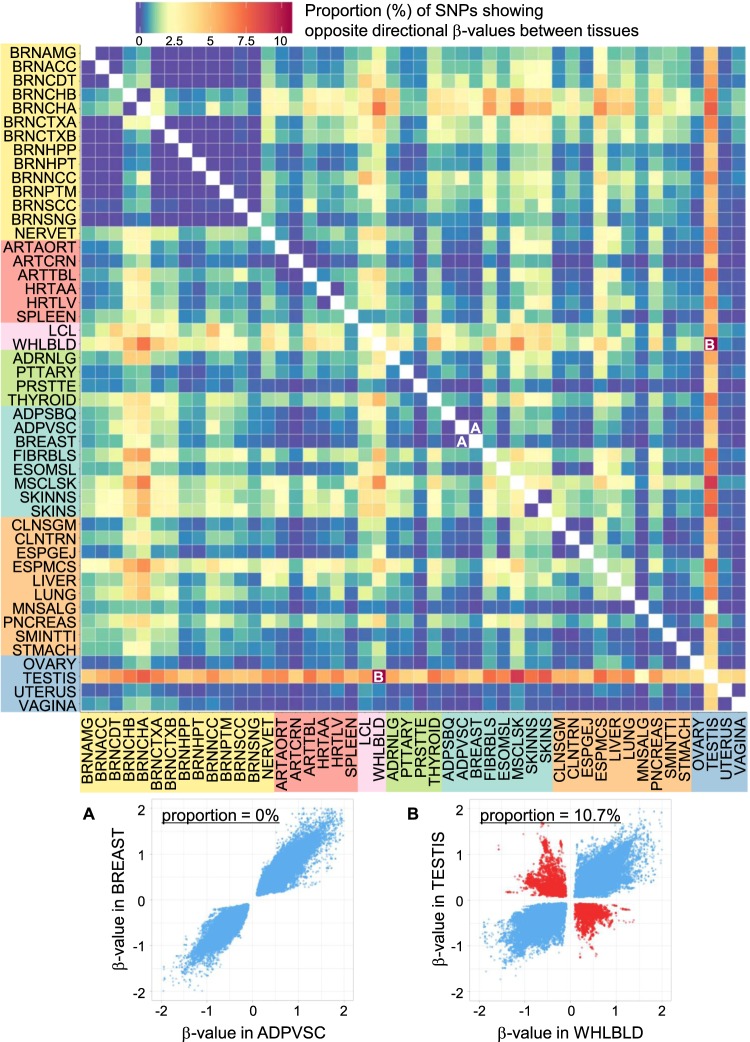
Heatmap of the proportion of SNPs showing opposite directional *β*-values. For each tissue pair, SNPs showing significant eQTL signals in both tissues were extracted. Direction of the *β*-values (effect sizes) of the SNPs was compared in each tissue pair, and proportion (%) of the SNPs that showed opposite directional *β*-values is shown by heatmap with the color scale shown on the top (blue to red). Plots of *β*-values of the SNPs in the two representative tissue pairs are shown on the bottom: **A** visceral adipose (ADPVSC) vs. breast (BREAST) and **B** whole blood (WHLBLD) vs. testis (TESTIS). SNPs with the same directional *β*-values are indicated as blue points, and SNPs with the opposite directional *β*-values are indicated as red points. There were no opposite eQTL effects found between ADPVSC and BREAST, while the largest proportion (10.7%) of the opposite eQTL effects were detected between WHLBLD and TESTIS

For each tissue pair in the 48 tissues (1128 pairs in total), the SNPs that showed eQTL signals in both tissues of the pair were plotted with respect to the *β*-value. The summary of the proportion of the SNPs showing the opposite directional *β*-values in the tissue pair is shown by heatmap in Fig. 3. Two representative plots are shown for (**A**) visceral adipose (ADPVSC) vs. breast (BREAST), in which no opposite eQTL effects were detected, and for (**B**) whole blood (WHLBLD) vs. testis (TESTIS), in which the largest proportion of the SNPs with opposite directional *β*-values (10.7%) was detected. As expected, testis showed high intensity of the opposite eQTL effects against almost all other tissues. Based on the averaged proportions between the organ system categories (Supplementary Table S3), the tissues in the same organ system category had a tendency to show relatively low intensity of the opposite eQTL effects, as remarkably shown in the brain tissues. In accordance with the results in Fig. 2, the cerebellar tissues showed clearly different intensity compared to the other brain tissues, which might indicate the well-documented, different gene expression pattern of cerebellum from other parts of the brain [21, 22].

Based on the proportions of SNPs showing opposite directional *β*-values, clustering analysis of the 48 tissue types was further performed. The result was depicted by heatmap in Supplementary Fig. S3. The brain tissues were well clustered with the cerebellar tissues apart as expected from Fig. 3. Although most of the organ system categories that we assigned for this study were not reproduced as a single cluster, some closely related tissue types were located in close positions such as LCL-WHLBLD, UTERUS-VAGINA, and LIVER-PNCREAS. Liver and pancreas both arise from the foregut endoderm in the embryonic development and possibly share a common progenitor population [23], whose common gene regulation pattern might be related to the similar opposite eQTL effect fractions against other tissue types.

### Biological properties of SNPs with the opposite eQTL effects

**Fig. 4:**
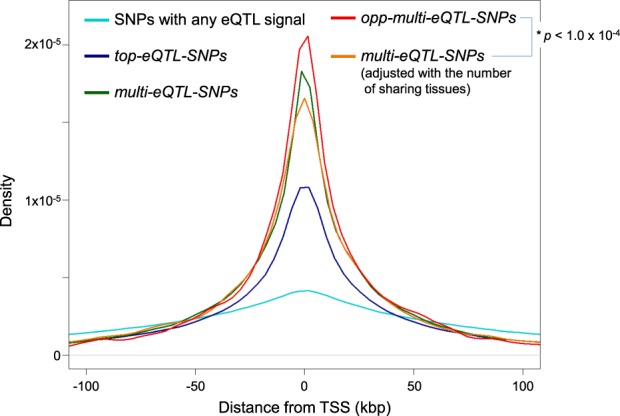
Distribution of the distance from TSS in each type of SNPs. For each type of SNPs (SNPs with any eQTL signal, *top-eQTL-*, *multi-eQTL-*, and *opp-multi-eQTL-SNPs*), the distribution of the distance from TSS is shown (in light blue, dark blue, green, and red, respectively). To adjust the number of sharing tissues of *multi-eQTL-SNPs* to that of *opp-multi-eQTL-SNPs*, subset of the *multi-eQTL-SNPs* was also analyzed, which is shown in an orange line. The TSS enrichment was evaluated on kurtosis of the density plot, and its significance level in *opp-multi-eQTL-SNPs* compared to adjusted *multi-eQTL-SNPs* was *p* < 1.0 × 10^−4^, which was determined by 10,000-time random sampling

The distribution of the distance from TSS in each type of SNPs (SNPs with any eQTL signal, *top-eQTL-SNPs*, *multi-eQTL-SNPs*, and *opp-multi-eQTL-SNPs*) is shown in Fig. 4. The distribution of the *top-eQTL-SNPs* was enriched at the TSS compared to all SNPs with any eQTL signal, which is in accordance with the common knowledge of eQTLs that the statistical significance of eQTLs becomes higher when it is close to the TSS [24–26]. The distribution of the *multi-eQTL-SNPs* was more enriched at the TSS than that of the *top-eQTL-SNPs*. This is also in consistence with another report on eQTLs using multiple types of tissues, in which the eQTLs shared by multiple tissues were located closer to the TSS than the eQTLs uniquely detected in a single tissue [27]. Interestingly, even more enrichment at the TSS was observed with the *opp-multi-eQTL-SNPs* (density ratio at TSS = 1.4 between *multi-eQTL-SNPs* and *opp-multi-eQTL-SNPs*). There could be a possibility that this enrichment at the TSS was plausibly caused by the increase of the number of sharing tissues rather than the opposite eQTL effects. To reject this hypothesis, the *multi-eQTL-SNPs* were thus randomly subsampled so that the number of sharing tissues was adjusted with that of the *opp-multi-eQTL-SNPs*. The distribution of the adjusted *multi-eQTL-SNPs* showed significantly less enrichment at the TSS compared to the *opp-multi-eQTL-SNPs* (*p* *<* 1.0 × 10^−4^), while it was not significantly different from that of the *multi-eQTL-SNPs*.

The opposite eQTL effects could be caused by epigenetically altered gene regulation patterns between tissues. To investigate a possible participation of epigenetic factors, we referred to histone modification (H3K4me1, H3K4me3, H3K9ac, and H3K27ac) and DNase sensitivity annotation data from the Roadmap Epigenomics project on up to 127 different cell lines [6], which are included in the HaploReg dataset [19]. For each cell line, the fractions of SNPs with each epigenetic annotation were calculated for *multi-eQTL-SNPs* and *opp-multi-eQTL-SNPs*. Since the enrichment at TSS in the *opp-multi-eQTL-SNPs* can be a confounding factor, the fractions were also calculated for a subset of the *multi-eQTL-SNPs* (TSS-distance-adjusted *multi-eQTL-SNPs*), which was generated by random subsampling so that the null distribution of the TSS distance was matched with that of the *opp-multi-eQTL-SNPs*. As shown in Supplementary Table S4, the mean fractions across cell lines were small in *multi-eQTL-SNPs* and large in *opp-multi-eQTL-SNPs* in all of the epigenetic annotations, which were statistically significant (*p* < 0.05) compared to the fractions in the TSS-distance-adjusted *multi-eQTL-SNPs*. Therefore, the epigenetic annotation enrichment can be related not only to the locational enrichment at TSS, but also to the opposite eQTL effects, which might be a molecular basis to regulate gene expression in the opposite directions between tissues.

### Association of opposite eQTL effects with complex traits

Based on the above analyses, the *opp-multi-eQTL-SNPs* showed relationship with the difference of the gene regulation pattern among tissues, according to the proximity in the distance from TSS and the possible involvement of epigenetic factors. To further evaluate the biological importance of the opposite eQTL effects, the LD relationship between each type of eQTL SNPs and the complex trait-associated SNPs reported in the GWAS catalog (*GWAS-SNPs*) was assessed. As summarized in Table 2, the proportion of the eQTL SNPs in LD with the *GWAS-SNPs* (*r*^*2*^ > 0.8) increased in the order of SNPs with any eQTL signal, *top-eQTL-SNPs*, *multi-eQTL-SNPs*, and *opp-multi-eQTL-SNPs*. Surprisingly, one out of four *opp-multi-eQTL-SNPs* (2,498 out of 9,290; 26.9%) was in LD with the *GWAS-SNPs*.

**Table 2:** Counts and proportions of the SNPs in LD with *GWAS-SNPs*

Type of SNPs	Adjustment	Total	LD with *GWAS-SNPs* (*r*^2^ > 0.8)	Proportion (%)
SNPs with any eQTL signal	–	2,842,590	322,981	11.4
*top-eQTL-SNPs*	–	210,878	27,054	12.8
*multi-eQTL-SNPs*	–	101,621	20,548	20.2
	TSS distance^a^	–	–	20.5 [19.8, 21.3]
*opp-multi-eQTL-SNPs*	–	9,290	2,498	26.9^b^

^a^Distribution of TSS distance in *multi-eQTL-SNPs* was adjusted on that in *opp-multi-eQTL-SNPs* by random sampling. The sampling was repeated by 10,000 times, and the mean is shown with 95% confidence interval in parentheses

^b^Significance levels of the proportion enrichment were *p* *<* 1.0 × 10^−15^ compared to the non-adjusted *multi-eQTL-SNPs* (Fisher’s exact test) and *p* *<* 1.0 × 10^−4^ compared to the adjusted *multi-eQTL-SNPs* (random sampling repeated by 10,000 times)

The enrichment with the *GWAS-SNPs* could be caused because the location of the SNPs was enriched at the TSS as reported in other studies [28, 29], rather than because the SNPs showed the opposite eQTL effects. To reject this possibility, the same analysis was conducted on the TSS-distance-adjusted *multi-eQTL-SNPs*. As a result, the proportion of the SNPs in LD with the *GWAS-SNPs* was not significantly different between the adjusted and non-adjusted *multi-eQTL-SNPs* (20.5% vs. 20.2%), while the proportion in the *opp-multi-eQTL-SNPs* was significantly higher than that in the adjusted *multi-eQTL-SNPs* (*p* *<* 1.0 × 10^−4^). These support that the enrichment of the *GWAS-SNPs* in the *opp-multi-eQTL-SNPs* was attributed to their own functional effects rather than the distance from the TSS. Since replication tendency of SNPs could be another confounding factor (i.e., *opp-multi-eQTL-SNPs* might be more likely to replicate in eQTL studies), we investigated the replication rates of TSS-distance-adjusted *multi-eQTL-SNPs* and *opp-multi-eQTL-SNPs* in other 12 independent eQTL datasets (Supplementary Table S5); however, there was no clear replication difference, i.e., while six datasets favored *opp-multi-eQTL-SNPs*, the other six ones favored TSS-distance-adjusted *multi-eQTL-SNPs*. Although not all confounding factors were excluded, the opposite eQTL effects could be a factor underlying the development of complex traits in GWASs.

**Fig. 5:**
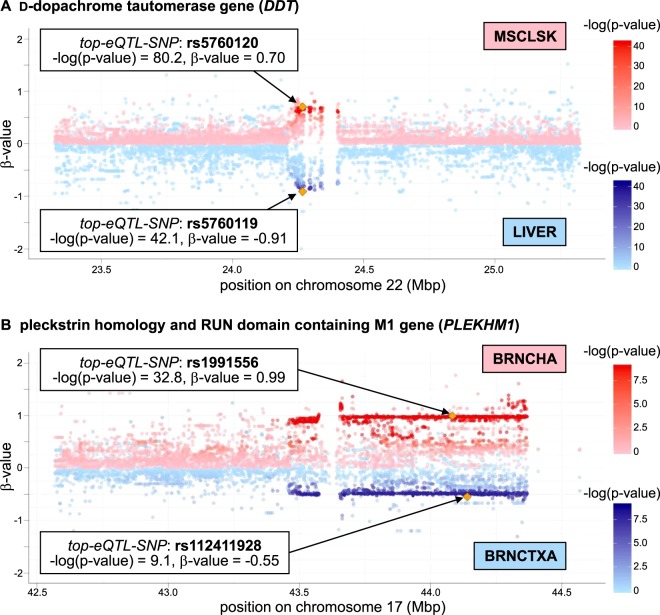
Distribution of the *β*-value and *p*-value of GWAS-related SNPs with opposite eQTL effects. The *opp-multi-eQTL-SNPs* that are in LD (*r*^*2*^ > 0.8) with the *GWAS-SNPs* were extracted, and examples of the genes affected by such GWAS-related *opp-multi-eQTL-SNPs* were selected. Distribution of *β*-value (*y*-axis) and negative logarithm of *p*-value (color scale) of all tested SNPs within 1 Mbp distance from TSS of the selected gene is shown with respect to the position on chromosome. **A** d-Dopachrome tautomerase gene (*DDT* [MIM: 602750]). Plots for skeletal muscle (MSCLSK) and liver (LIVER) are shown in red and blue, respectively. **B** Pleckstrin homology and RUN domain containing M1 gene (*PLEKHM1* [MIM: 611466]). Plots for cerebellum (BRNCHA) and brain cortex (BRNCTXA) are shown in red and blue, respectively

Examples of the distribution of *β*-value and *p*-value of the GWAS-related *opp-multi-eQTL-SNPs* with respect to the position on chromosome are shown in Fig. 5. The opposite eQTL effects on d-dopachrome tautomerase gene (*DDT* [MIM: 602750]) between blood and liver tissues was previously reported [12]. The same opposite eQTL effects were recapitulated in this study, and also some other tissues such as skeletal muscle (MSCLSK) showed the opposite direction of eQTL effects as shown in Fig. 5**A**, in which the *opp-multi-eQTL-SNPs* (rs5760120 and rs5760119) was in LD with rs2739330, reported in the association study of the liver enzyme level trait [30].

As discussed above, the cerebellar tissues showed more opposite eQTL effects than other parts of the brain. Interestingly, there were GWAS-related opposite eQTL effects detected between the cerebellar tissues and other brain tissues. An example of such cases is shown in Fig. 5**B**, in which the SNPs showed opposite eQTL effects on pleckstrin homology and RUN domain containing M1 gene (*PLEKHM1* [MIM: 611466]) between cerebellum (BRNCHA) and brain cortex (BRNCTXA). The identified opp-*multi-eQTL-SNPs* (rs1991556 and rs112411928) were in LD with the SNPs reported in neurological diseases such as Parkinson disease (PD [MIM: 168600]) [31–33], progressive supranuclear palsy ([MIM: 601104]) [34], and corticobasal degeneration [35]. The susceptibility difference between brain regions including cerebellum found in those diseases could be related to the difference of the gene regulation pattern, which might have been detected as the opposite eQTL effects in this study.

## Discussion

The largest eQTL database from the GTEx project provided an ideal dataset to analyze the opposite eQTL effects between tissues, resulting in several findings to be noted. First, the analysis of the diverse tissue pairs revealed that the genes affected by the opposite eQTL effects (*opp-multi-eQTL-Genes*) can be discovered more frequently than expected from previous eQTL studies (Table 1). Second, the statistics of the opposite eQTL effects suggested that the indices relating to the *opp-multi-eQTL-Genes* and *SNPs* (i.e., the proportion of *opp-multi-eQTL-Genes* in *multi-eQTL-Genes* shown in Fig. 2, and the proportion of SNPs with opposite directional β-values shown in Fig. 3) could be used as an indicator of the different gene regulation pattern compared to other tissues in a study. Finally, the enrichment analysis of the *opp-multi-eQTL-SNPs* about the distance from TSS, the epigenetic annotations, and the *GWAS-SNPs* showed their possible involvement in altered gene regulation depending on tissue types and association with the complex traits in GWASs (Fig. 4, Table S4, and Table 2), which supported the importance of the opposite eQTL effects as the biologically meaningful annotations of genes and genetic variants.

In this study, the summary statistics-based analysis method evaluated the discordance of eQTL effects between different tissues by comparing the direction of the primary, or most significant, eQTL signals with the LD relationship taken into account. Our method might have effectively worked to remove less meaningful opposite eQTL effects. As an example in Supplementary Fig. S4, the eQTL effects on solute carrier family 37 member 1 gene (*SLC37A1* [MIM: 608094]) can be considered to be in the opposite direction between pituitary (PTTARY) and skeletal muscle (MSCLSK), if the discordance is evaluated by all (including secondary) eQTL signals or by the primary eQTL signals without LD calculation. In this case, the *top-eQTL-SNPs* in the two tissues (rs4919992 for PTTARY and rs228048 for MSCLSK) are localized in the different LD blocks (i.e., *r*^*2*^ < 0.8). rs228048 was significant only in MSCLSK, while rs4919992 showed significant eQTL signals in both tissues. However, rs4919992 apparently composes the secondary marginal eQTL signals in MSCLSK, which are independent from the primary eQTL signals tagged by rs228048. This kind of discordance would be less meaningful to describe the gene regulation difference between the tissues, compared to the discordance detected between the *top-eQTL-SNPs* in LD. Consistently, rs4919992 and rs228048 did not show the LD relationship with the *GWAS-SNPs*. The hit rate of *GWAS-SNPs* in the *opp-multi-eQTL-SNPs* was 26.9% (Table 2), but it significantly dropped to 20.7% when no LD threshold was set to define the *opp-multi-eQTL-SNPs*, which supports that the LD evaluation of the primary eQTLs in this study was effective for assessing the discordance of eQTL effects between tissues.

In conclusion, as the tissue-dependent discordant eQTL effects, the opposite eQTL effects were discovered for the significant proportion of the *eQTL-Genes* in the GTEx database (2,323 out of 31,212; 7.4%). Such opposite eQTL effects were shown to be associated with the complex traits in GWASs. Based on these analyses, it appears that the frequency of the opposite eQTL effects has been underestimated, and they would be a rather common phenomenon, which possibly play an important role in the tissue-specific gene regulation influencing the development of complex traits. Therefore, the association with the opposite eQTL effects can be a biologically meaningful annotation of genes and genetic variants to further understand the results of GWASs. Although the GTEx database is currently the largest eQTL database, it is not yet a complete dataset for all tissues in human, and the diversity of the populations is also limited. Since this study was based on the eQTL results analyzed for each single tissue, meta-analysis of eQTL effects across tissue types could be useful to discover true opposite eQTL effects in the multi-tissue dataset [36]. As the future perspective, incorporating other tissue types such as immune cells and developing a meta-analysis method suitable for multi-tissue eQTL studies will be important for the purpose of revealing the whole image of the opposite eQTL effects and their contribution to the genetics of complex traits.

### Web resources

GTEx Portal: https://www.gtexportal.org/

GWAS Catalog: https://www.ebi.ac.uk/gwas/

1000 Genomes Project: http://www.internationalgenome.org/

PLINK 1.9: https://www.cog-genomics.org/plink2

HaploReg v4.1: https://pubs.broadinstitute.org/mammals/haploreg/haploreg.php

## Supplementary information

Supplementary files
