# Supplementary material for: Biological characterization of expression quantitative trait loci (eQTLs) showing tissue-specific opposite directional effects
Source: Eur J Hum Genet. 2019 Jul 11;27(11):1745–56. doi: 10.1038/s41431-019-0468-4 (PMC6871526; doi:10.1038/s41431-019-0468-4)
Supplement: Supplementary file 1 — Supplementary files [file 41431_2019_468_MOESM1_ESM.pdf]

For a given *eQTL-Gene*, between tissue-x and tissue-y:

**analysis table**	tissue-x	tissue-y
SNP-x<br>*top-eQTL-SNP* in tissue-x	β_{xx}	β_{xy}
SNP-y<br>*top-eQTL-SNP* in tissue-y	β_{yx}	β_{yy}

β: effect size of eQTL signal.

If SNP-x(y) is not significantly active in tissue-y(x), β_{xy} (β_{yx}) is set as 0.

35,207,136 analysis tables (**31,212** *eQTL-Genes*, **1,128** tissue pairs from 48 tissues)

The given *eQTL-Gene* has significant eQTL SNP(s) in both tissues ($\beta_{xx} \neq 0 \cap \beta_{yy} \neq 0$)

3,540,453 analysis tables

At least one of SNP-x and SNP-y is significant in both tissues ($\beta_{xy} \neq 0 \cup \beta_{yx} \neq 0$)

2,488,564 analysis tables

**Number of analysis tables**		Direction difference of eQTL effects (sign of $\beta_{xx}\times\beta_{xy}$ and $\beta_{yx}\times\beta_{yy}$)<br>Opposite<br>(-/-, -/0, 0/-)	 Others<br>(+/+, +/0, 0/+, +/-, -/+)
LD between SNP-x and SNP-y	$r^2 > 0.8$	**1a**: **15,583**	**2a**: **1,570,689**
	$r^2 \leq 0.8$	**1b**: **27,386**	**2b**: **874,906**

genes and SNPs in group **1a** or **2a** ⇒ *multi-eQTL-Genes* and *multi-eQTL-SNPs*
genes and SNPs in group **1a** ⇒ *opp-multi-eQTL-Genes* and *opp-multi-eQTL-SNPs*

Figure S1. Scheme to analyze direction of eQTL effects between different tissues.
An analysis table was made for each *eQTL-Gene* between each tissue pair (35,207,136 analysis tables in total). SNP-x is *top-eQTL-SNP* of the given *eQTL-Gene* in tissue-x. β_{xy} is effect size of SNP-x in tissue-y (if SNP-x is not significantly detected in tissue-y, β_{xy} was set as 0). Only the cases that the given *eQTL-Gene* has SNP(s) with significant eQTL signal in both of the tissue pair (i.e., $\beta_{xx} \neq 0 \cap \beta_{yy} \neq 0$) were moved into the next analysis (3,540,453 analysis tables). When at least one of SNP-x and SNP-y is significant in both tissues (i.e., $\beta_{xy} \neq 0 \cup \beta_{yx} \neq 0$), the direction difference of the eQTL effects between the tissue pair can be discussed. Therefore, the cases of (i) SNP-x and SNP-y are identical (i.e., $\beta_{xx} = \beta_{yx}$ and $\beta_{xy} = \beta_{xy}$) and (ii) SNP-x and SNP-y are not identical but at least one of them is significant in both tissues were selected (2,488,564 analysis tables). The direction difference of the eQTL effects between the tissue pair was determined by the sign of $\beta_{xx}\times\beta_{xy}$ and $\beta_{yx}\times\beta_{yy}$, and the cases of $\beta_{xx}\times\beta_{xy} \leq 0 \cup \beta_{yx}\times\beta_{yy} \leq 0$ were considered as opposite direction. By addition of LD evaluation between SNP-x ad SNP-y (r^2 cutoff is 0.8. If SNP-x and SNP-y are identical, r^2 was set as 1), the analysis tables were divided into four groups (**1a**, **1b**, **2a**, and **2b**). The *eQTL-Genes* and *top-eQTL-SNPs* in group **1a** or **2a** were designated as *multi-eQTL-Genes* and *multi-eQTL-SNPs*, and those in group **1a** were designated as *opp-multi-eQTL-Genes* and *opp-multi-eQTL-SNPs*.

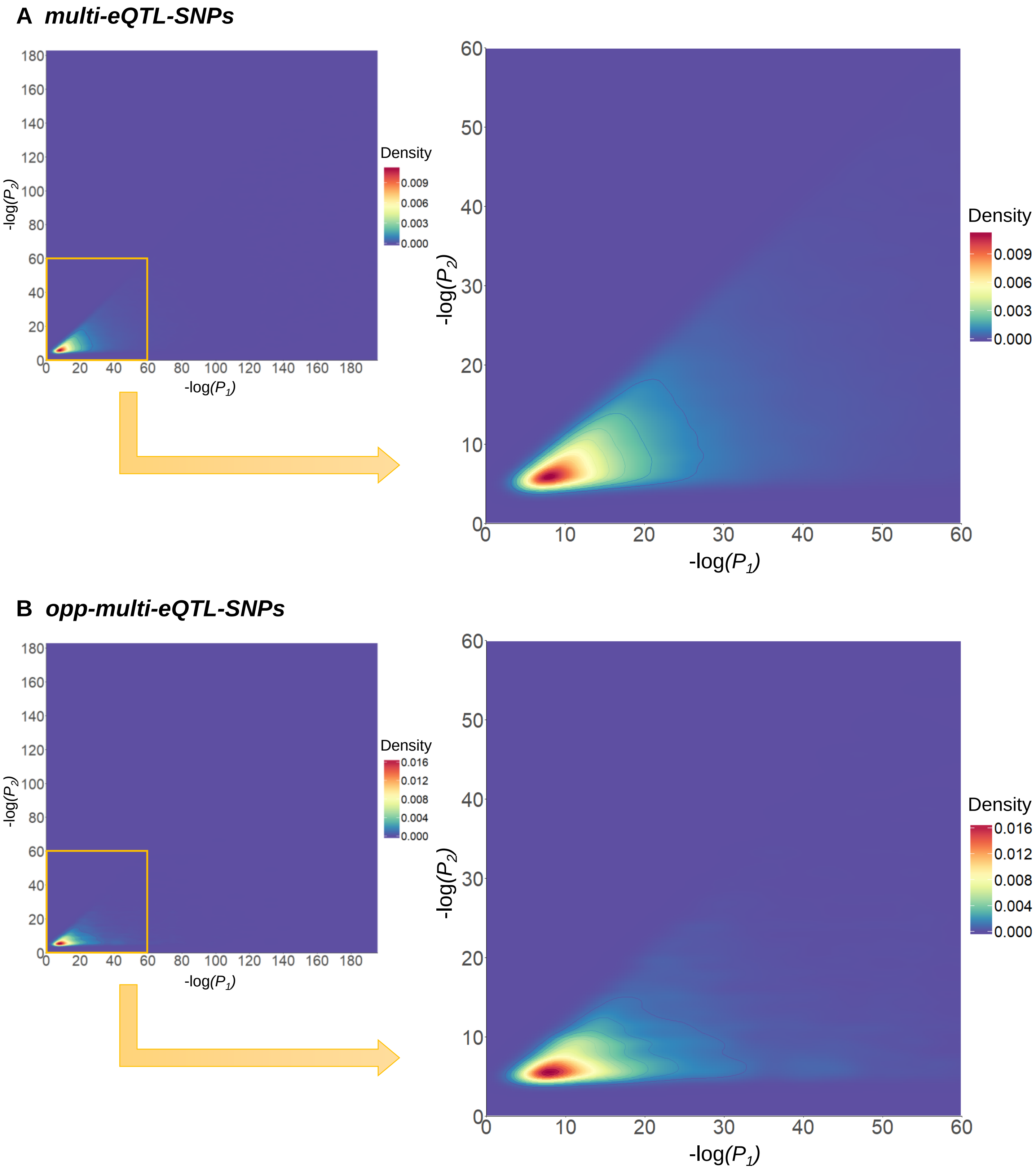

Figure S2. 2D density map of negative logarithm of p-value_1 and 2. Of the two p-values in each analyzed tissue pair, p-value_1 and 2 were designated as lower and higher p-values, respectively. 2D density map is depicted for the eQTL effects of *multi-eQTL-SNPs* (**A**), and *opp-multi-eQTL-SNPs* (**B**). The difference of the density between them is also shown (**C**).

C Density difference between *multi-eQTL-SNPs* and *opp-multi-eQTL-SNPs*

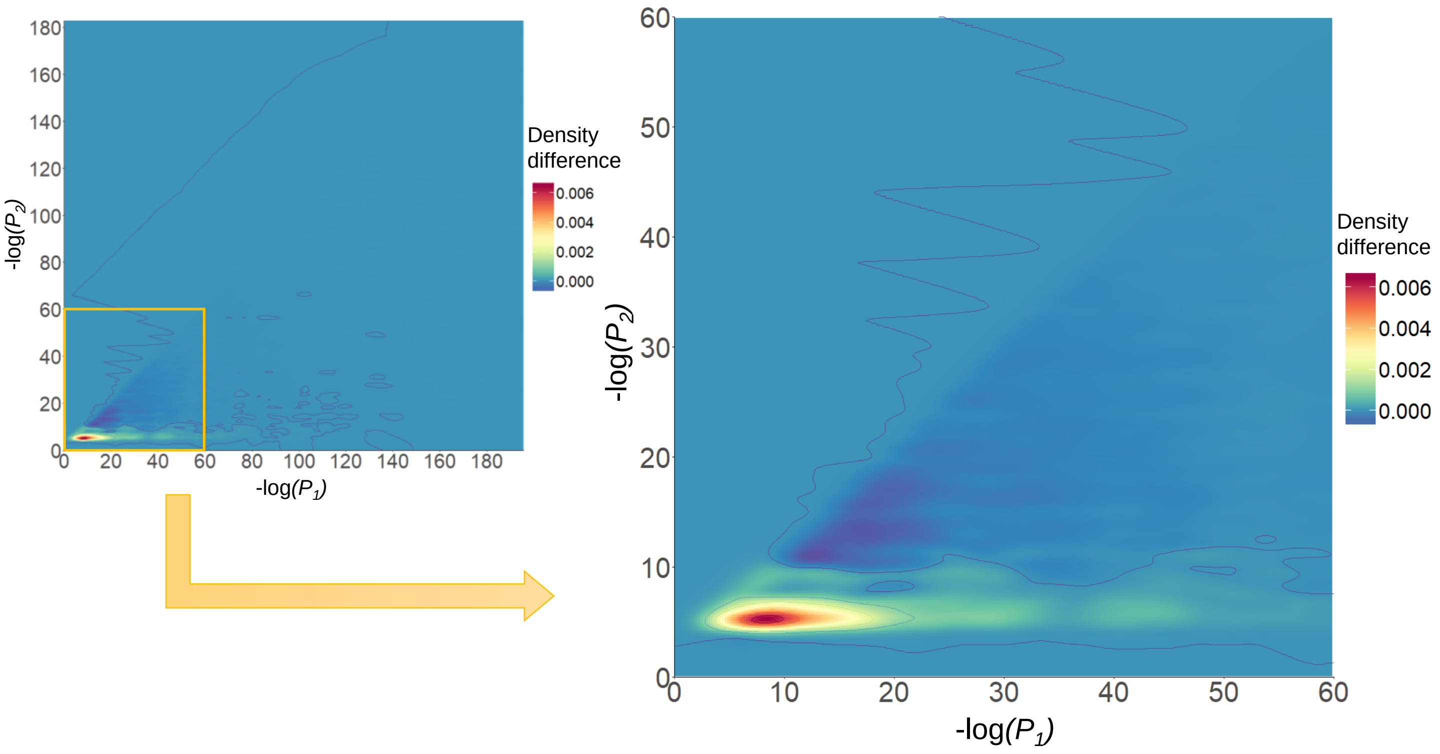

Figure S2. (continued)

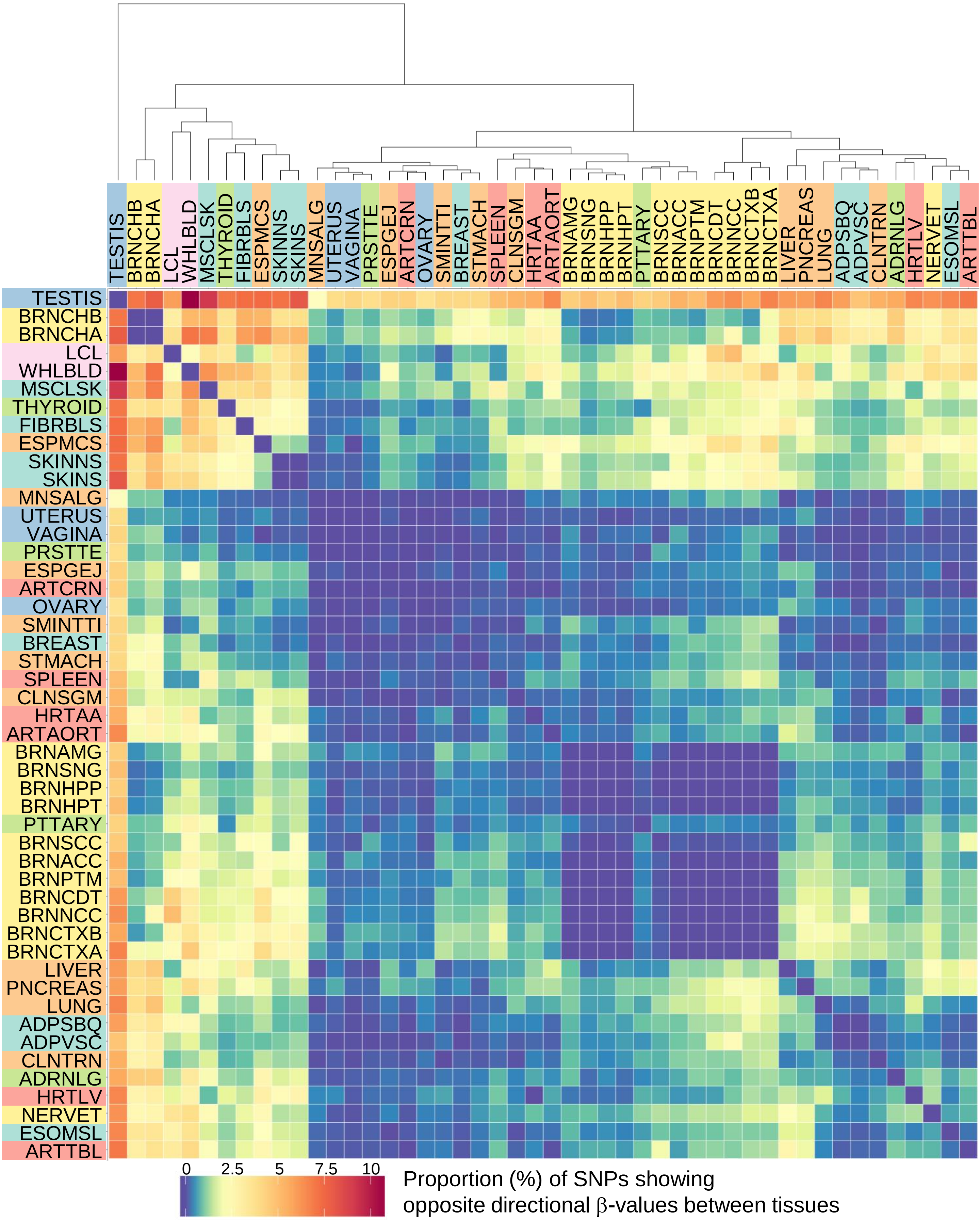

Figure S3. Clustering analysis of tissue types based on the proportions of SNPs showing opposite directional β-values between tissues.

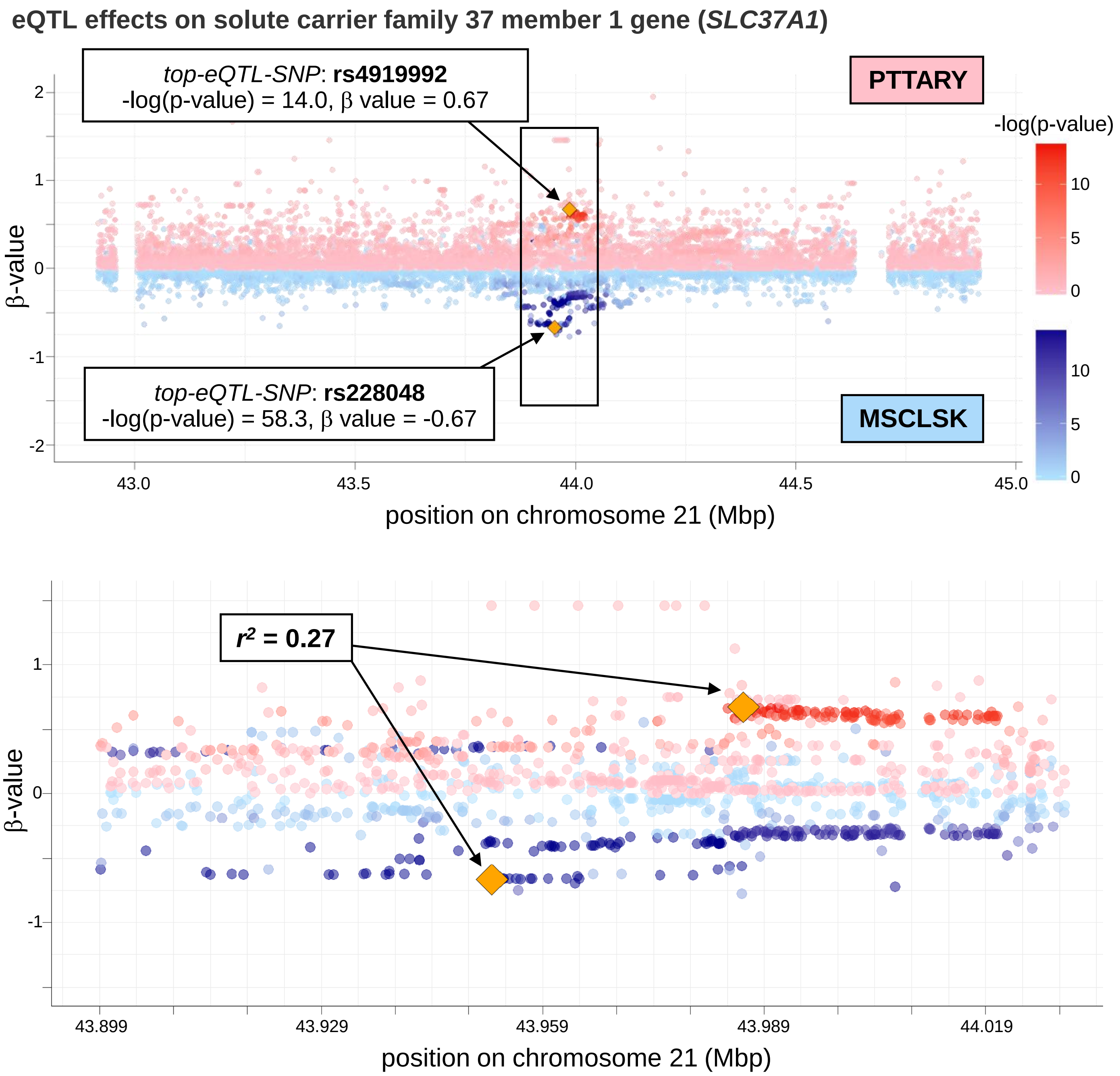

Figure S4. Example of less meaningful opposite eQTL effects.
Distribution of the β-value and negative logarithm of p-value of all tested SNPs within 1Mbp distance from TSS of solute carrier family 37 member 1 gene (*SLC37A1*) is shown with respect to the position on chromosome. The lower panel is the enlarged view of the square part in the upper panel. The eQTL effects on *SLC37A1* can be considered in the opposite direction between pituitary (PTTARY) and skeletal muscle (MSCLSK), if the discordance is evaluated by all (including secondary) eQTL signals or by the primary eQTL signals without LD calculation. However, the *top-eQTL-SNPs* in the two tissues (rs4919992 for PTTARY and rs228048 for MSCLSK) are localized in the different LD blocks ($r^2 < 0.8$). rs228048 was significant only in MSCLSK. Although rs4919992 was significant in both tissues, it composes the secondary marginal eQTL signals in MSCLSK, which are independent from the far more significant primary eQTL signals tagged by rs228048. Therefore, it is dubious to consider this kind of eQTL effects as the meaningful opposite effects between different tissues.

Table S1. Detailed description of SNPs based on the reference genome sequence (GRCh37) for the rsIDs that appeared in the main text.

dbSNP rsID	Sequence name	Change
rs1991556	GRCh37.p13 chr 1	NC_000001.10:g.797440T>C
rs112411928	GRCh37.p13 chr 17	NC_000017.10:g.44139762C>G
rs2739330	GRCh37.p13 chr 22	NC_000022.10:g.24295286T>C
rs228048	GRCh37.p13 chr 21	NC_000021.8:g.43952141T>C
rs4919992	GRCh37.p13 chr 21	NC_000021.8:g.43986175C>T
rs5760120	GRCh37.p13 chr 22	NC_000022.10:g.24266867G>A
rs5760119	GRCh37.p13 chr 22	NC_000022.10:g.24266831G>C

Table S2. Abbreviations and organ system categories assigned for 48 tissues.

Tissue Name	Abbreviation	Organ System Category
Brain_Amygdala	BRNAMG	Nervous System
Brain_Anterior_cingulate_cortex_BA24	BRNACC	Nervous System
Brain_Caudate_basal_ganglia	BRNCDT	Nervous System
Brain_Cerebellar_Hemisphere	BRNCHB	Nervous System
Brain_Cerebellum	BRNCHA	Nervous System
Brain_Cortex	BRNCTXA	Nervous System
Brain_Frontal_Cortex_BA9	BRNCTXB	Nervous System
Brain_Hippocampus	BRNHPP	Nervous System
Brain_Hypothalamus	BRNHPT	Nervous System
Brain_Nucleus_accumbens_basal_ganglia	BRNNCC	Nervous System
Brain_Putamen_basal_ganglia	BRNPTM	Nervous System
Brain_Spinal_cord_cervical_c-1	BRNSCC	Nervous System
Brain_Substantia_nigra	BRNSNG	Nervous System
Nerve_Tibial	NERVET	Nervous System
Artery_Aorta	ARTAORT	Cardiovascular/Lymphatic System
Artery_Coronary	ARTCRN	Cardiovascular/Lymphatic System
Artery_Tibial	ARTTBL	Cardiovascular/Lymphatic System
Heart_Atrial_Appendage	HRTAA	Cardiovascular/Lymphatic System
Heart_Left_Ventricle	HRTLV	Cardiovascular/Lymphatic System
Spleen	SPLEEN	Cardiovascular/Lymphatic System
Cells_EBV-transformed_lymphocytes	LCL	Hematopoietic Cells
Whole_Blood	WHLBLD	Hematopoietic Cells
Adrenal_Gland	ADRNLG	Endocrine System
Pituitary	PTTARY	Endocrine System
Prostate	PRSTTE	Endocrine System
Thyroid	THYROID	Endocrine System
Adipose_Subcutaneous	ADPSBQ	Integumentary/Musculoskeletal System
Adipose_Visceral_Omentum	ADPVSC	Integumentary/Musculoskeletal System
Breast_Mammary_Tissue	BREAST	Integumentary/Musculoskeletal System
Cells_Transformed_fibroblasts	FIBRBLS	Integumentary/Musculoskeletal System
Esophagus_Muscularis	ESOMSL	Integumentary/Musculoskeletal System
Muscle_Skeletal	MSCLSK	Integumentary/Musculoskeletal System
Skin_Not_Sun_Exposed_Suprapubic	SKINNS	Integumentary/Musculoskeletal System
Skin_Sun_Exposed_Lower_leg	SKINS	Integumentary/Musculoskeletal System
Colon_Sigmoid	CLNSGM	Gastrointestinal/Respiratory System
Colon_Transverse	CLNTRN	Gastrointestinal/Respiratory System
Esophagus_Gastroesophageal_Junction	ESPGEJ	Gastrointestinal/Respiratory System
Esophagus_Mucosa	ESPMCS	Gastrointestinal/Respiratory System
Liver	LIVER	Gastrointestinal/Respiratory System
Lung	LUNG	Gastrointestinal/Respiratory System
Minor_Salivary_Gland	MNSALG	Gastrointestinal/Respiratory System
Pancreas	PNCREAS	Gastrointestinal/Respiratory System
Small_Intestine_Terminal_Ileum	SMINTTI	Gastrointestinal/Respiratory System
Stomach	STMACH	Gastrointestinal/Respiratory System
Ovary	OVARY	Genitourinary System
Testis	TESTIS	Genitourinary System
Uterus	UTERUS	Genitourinary System
Vagina	VAGINA	Genitourinary System

Table S3. Averaged proportions of SNPs showing opposite directional β-values between the organ system categories. (Minimum value in each row is highlighted in red.)

	Nerv	Card	Hem	Endo	Mus	Gast	Gen
**Nerv**	0.54	0.92	2.05	0.97	1.59	1.35	1.67
**Card**	0.92	0.34	1.48	0.63	0.95	0.73	1.55
**Hem**	2.05	1.48	2.77	1.91	2.26	1.54	2.55
**Endo**	0.97	0.63	1.91	0.44	1.14	0.76	1.40
**Mus**	1.59	0.95	2.26	1.14	1.30	1.09	1.86
**Gast**	1.35	0.73	1.54	0.76	1.09	0.63	1.51
**Gen**	1.67	1.55	2.55	1.40	1.86	1.51	1.95

Nerv, nervous system
Card, cardiovascular/lymphatic system
Hem, hematopoietic cells
Endo, endocrine system
Mus, integumentary/musculoskeletal system
Gast, gastrointestinal/respiratory system
Gen, genitourinary system

Table S4. Fractions of SNPs with epigenetic annotation in Roadmap Epigenomics database.

Epigenetic annotation	Total number of cell lines annotated	mean fraction of annotated SNPs across cell lines (%)		
		multi-eQTL-SNPs	TSS-distance-adjusted *multi-eQTL-SNPs* [a]	*opp-multi-eQTL-SNPs*
DNase	53	7.6	8.6 [8.2, 8.9]	9.6
H3K4me1	127	20.6	21.9 [21.4, 22.3]	24.0
H3K4me3	127	13.2	15.0 [14.5, 15.4]	15.7
H3K9ac	62	17.0	18.7 [18.3, 19.2]	20.0
H3K27ac	98	17.1	18.5 [18.1, 19.0]	19.8

(a) Distribution of TSS distance in *multi-eQTL-SNPs* was adjusted on that in *opp-multi-eQTL-SNPs* by random sampling. The sampling was repeated by 10,000 times, and the mean is shown with 95% confidence interval in parentheses.

Table S5. Replication rates (%) of the extracted SNPs in other eQTL datasets.

eQTL study [a]	TSS-distance-adjusted *multi-eQTL-SNPs* [b]	*opp-multi-eQTL-SNPs*
Montgomery et al, 2010	0.24	0.18
Schadt et al, 2008	0.39	0.47
Gibbs et al, 2010	1.12	1.66
Stranger et al, 2008	1.53	1.91
Li et al, 2014	0.91	0.76
Westra et al, 2013	22.2	24.1
Hao et al, 2012	2.02	1.71
Koopmann et al, 2014	0.99	1.26
Fairfax et al, 2014	0.24	0.17
Grundberg et al, 2011	0.51	0.48
Lappalainen et al, 2013	49.0	53.7
Ramasamy et al, 2014	1.60	1.03

(a) Annotation data for the listed eQTL studies were downloaded from the HaploReg v4.1 website: https://pubs.broadinstitute.org/mammals/haploreg/data/

(b) A subset of *multi-eQTL-SNPs*, randomly sampled so that the distribution of TSS distance was adjusted on that of *opp-multi-eQTL-SNPs*.
